# Is Anterior Cervical Discectomy and Fusion Superior to Corpectomy and Fusion for Treatment of Multilevel Cervical Spondylotic Myelopathy? A Systemic Review and Meta-Analysis

**DOI:** 10.1371/journal.pone.0087191

**Published:** 2014-01-28

**Authors:** Ying-Chao Han, Zhu-Qing Liu, Shan-Jin Wang, Li-Jun Li, Jun Tan

**Affiliations:** 1 Department of Spinal Surgery, East Hospital, Tongji University School of Medicine, Shanghai, China; 2 Department of Medical Oncology, Shanghai Tenth People's Hospital, Tongji University School of Medicine, Shanghai, China; University of Toronto, Canada

## Abstract

**Objective:**

Both anterior cervical discectomy with fusion (ACDF) and anterior cervical corpectomy with fusion (ACCF) are used to treat cervical spondylotic myelopathy (CSM), however, there is considerable controversy as to whether ACDF or ACCF is the optimal treatment for this condition. To compare the clinical outcomes, complications, and surgical trauma between ACDF and ACCF for the treatment of CSM, we conducted a meta-analysis.

**Methods:**

We conducted a comprehensive search in MEDLINE, EMBASE, PubMed, Google Scholar and Cochrane databases, searching for relevant controlled trials up to July 2013 that compared ACDF and ACCF for the treatment of CSM. We performed title and abstract screening and full-text screening independently and in duplicate. A random effects model was used for heterogeneous data; otherwise, a fixed effect model was used to pool data, using mean difference (MD) for continuous outcomes and odds ratio (OR) for dichotomous outcomes.

**Results:**

Of 2157 citations examined, 15 articles representing 1372 participants were eligible. Overall, there were significant differences between the two treatment groups for hospital stay (M = −5.60, 95% CI = −7.09 to −4.11), blood loss (MD = −151.35, 95% CI = −253.22 to −49.48), complications (OR = 0.50, 95% CI = 0.35 to 0.73) and increased lordosis of C2–C7 (MD = 3.70, 95% CI = 0.96 to 6.45) and fusion segments angles (MD = 3.38, 95% CI = 2.54 to 4.22). However, there were no significant differences in the operation time (MD = −9.34, 95% CI = −42.99 to 24.31), JOA (MD = 0.24, 95% CI = −0.10 to 0.57), VAS (MD = −0.06, 95% CI = −0.81 to 0.70), NDI (MD = −1.37, 95% CI  = −3.17 to 0.43), Odom criteria (OR = 0.88, 95% CI = 0.60 to 1.30) or fusion rate (OR = 1.17, 95% CI = 0.34 to 4.11).

**Conclusions:**

Based on this meta-analysis, although complications and increased lordosis are significantly better in the ACDF group, there is no strong evidence to support the routine use of ACDF over ACCF in CSM.

## Introduction

Cervical spondylotic myelopathy (CSM) is a clinically symptomatic condition caused by compression of the spinal cord due to degeneration. It is a significant cause of disability in the adult population [1]–[3], notably causing progressive degenerative changes in the cervical spine of patients over 55 years of age [4], [5]. CSM is a common cause of neurological morbidity, and can substantially decrease quality of life [6]. The principal indication for surgery for CSM is the development of progressive spinal cord type symptoms and signs [7]. Surgical treatment has been advocated for CSM by many authors, however, the optimal surgical approach remains controversial. Anterior, posterior and combined anterior and posterior surgical approaches for CSM have all been proposed and encouraged. Anterior approaches usually involve anterior cervical discectomy with fusion (ACDF) or anterior cervical corpectomy with fusion (ACCF), whereas posterior cervical canal decompression approaches typically involve laminoplasty and laminectomy. In terms of anterior procedures, there is considerable controversy as to which reconstruction technique is best after anterior cervical decompression. The anterior approach to the cervical spine was developed in the 1950s by Smith [8] and Cloward [9]. ACDF can decompress the anterior spinal cord, preserve the stability of the spinal column and is associated with a low prevalence of graft extrusion or migration. However, some authors argue that ACDF may not be the optimal surgical approach for CSM due to the risk of incomplete decompression, limited visual exposure, and a high rate of non-union due to graft–host interfaces [10], [11]. An alternate means of improving the fusion rate after multi-level decompression is the use of ACCF [12]. In addition to improving the fusion rate, ACCF also provides for a more extensive decompression and serves as a source for autografting. Unfortunately, ACCF is a more difficult spinal surgery to perform and is also associated with a higher incidence of complications, such as increased risk of damage to the spinal cord or nerve roots, excessive bleeding, graft displacement or extrusion, and others [13]–[15].

The results of previous studies comparing the clinical effects of ACDF to ACCF for the treatment of CSM vary considerably. It is still uncertain whether ACDF is safer and more effective than ACCF. Therefore, to clarify these ambiguous findings we performed a meta-analysis to compare ACDF with ACCF for the treatment of CSM.

## Methods

### 2.1 Search strategies

To assemble all of the relevant literature, a search of relevant systematic reviews on CSM in the Cochrane Library (Cochrane library 2013), observational cohort studies (with and without control groups), systematic reviews, and clinical trials was conducted in MEDLINE (1966 to July 2013), EMBASE (1974 to July 2013), PubMed (1966 to July 2013) and Google Scholar (1966 to July 2013). The following search terms were used: cervical spondylotic myelopathy, cervical spine, discectomy, corpectomy, cervical spondylosis, surgical decompression, spinal fusion, and complications, with various combinations of the operators “AND”, “NOT”, and “OR”. We restricted the language to English. References cited in relevant articles and reviews were checked to identify additional studies. The full search strategy is available upon request from the corresponding authors (**Wang and Tan**).

The quality of the studies was independently assessed by two authors (Han and Liu), and the level of agreement between them was recorded. The decision on whether to include an article was made by manual screening of titles and abstracts, followed by full-text screening by the same reviewers. If additional data or clarification were necessary, we contacted the study authors. Any disagreements between reviewers were resolved by discussion with another reviewer (**Wang**).

### 2.2 Eligibility criteria

Studies were included if they met the following criteria: (1) adult patients over 18 years of age of both genders with CSM; (2) randomized or non-randomized controlled clinical studies; (3) studies compared ACDF with ACCF for treatment of CSM; (4) post-operative follow-up with included patients was for a minimum of 24 months; and (5) outcome assessment was based on the primary and secondary outcomes. The primary outcome was defined as major surgical complications, radiographic outcomes, fusion rate, or patient-related outcome measures regarding pain and quality of life using various validated questionnaires, such as the Japanese Orthopedic Association score (JOA), the Visual Analogue Score (VAS), the Neck Disability Index (NDI), and Odom criteria, among others. The secondary outcome included surgical data, such as the operation time, blood loss and length of hospital stay.

### 2.3 Exclusion criteria

Studies were excluded if they (1) had an average follow-up time of less than 24 months; (2) were uncontrolled; (3) described case reports or were systematic reviews; (4) dealt only with combined ACDF and ACCF surgery versus ACDF or ACCF alone for treatment of CSM.

### 2.4 Data extraction and management

Data were extracted independently by two reviewers (Han and Liu). Discussions were conducted to deal with disagreements, and when necessary, discussions included another independent expert (**Wang**). The following information was collected from each study: (1) general characteristics, including the authors, the year of publication, sample size, age, gender, duration of follow-up and the type of graft (*Table 1*); and (2) details of the clinical outcome measurement: the length of hospital stay, blood loss, operation time, JOA, VAS, NDI, Odom criteria, fusion rate, Cobb angles of C2–C7, segmental angle complications, and the type of complications, such as dysphagia, hoarseness, C5 palsy, infection, cerebral fluid leakage, donor site pain, epidural hematoma, graft related and hardware-related complications (*Tables 2 and 3*). All included studies met the inclusion and exclusion criteria. The extracted data were rechecked for accuracy or against the inclusion criteria by **Wang**.

**Table 1:** Characteristics of the included studies.

Study	Publition year	Sample Size		Mean age(years)		Sex(male/female)		Followup(months,years)		Graft	
		ACDF	ACCF	ACDF	ACCF	ACDF	ACCF	ACDF	ACCF	ACDF	ACCF
Li et al.[22]	2013	47	42	51.3±6.5		58M/31F		79.6±20.5m		Autograft,cage	Autograft,cage
Liu et al.[21]	2012	69	39	46.1±6.8	47.8±6.4	39M/30F	26M/13F	26.8m(12–29m)	26.4m(12–37m)	Cages,Atlantis plate	Titanium mesh cage,Atlantis plate
Kyung et al.[23]	2012	25	15	50.3±7.5	54.1±9.8	19M/6F	11M/4F	87.3±21.7m	94.3±25.3m	Autograft and cage	Autograft
Lin et al.[24]	2012	57	63	58.74±9.7	57.90±9.0	38M/19F	43M/20F	24m	24m	Autograft,cages, semi-constrained plating systems	Autograft,TMC, semi-constrained plating systems
Guo et ai.[25]	2011	43	24	52.7±9.4	55.2±10.1	24M/19F	13M/11F	37.7±7.2m	37.3±7.3m	Autograft and PEEK cages	Autograft and Titanium cage
Park et al.[26]	2010	45	52	49.3±9.7	49.4±8.7	28M/17F	22M/30F	25.7±6.2m	23.3±6.6m	Allograft	Allograft
Oh et al.[10]	2009	14	17	54.45±11.6		16M/15F		26.23±15.0m		Cage, allograft	Autograft
Uribe et al.[27]	2009	42	38	46.2	50.0	21M/21F	21M/17F	2.3 years	2.2 years	Titanium mesh cages,autograft	Titanium mesh cages,autograft
Hwang et al.[28]	2007	27	35	54.2	52.2	13M/14F	19M/16F	24m	24m	Titanium mesh cages,autograft	Titanium mesh cages,autograft
Nirala et al.[29]	2004	69	132	55	44	40M/29F	80M/52F	54m	48m	Autograft	Autograft
Hilibrand et al.[30]	2002	131	59	53	58	66M/65F	30M/29F	57m	73m	Autograft	Autograft
Jeffrey et al.[31]	2001	32	20	51.5(17–80)		27M/25F		3.6 years (2-7Y)		Allograft	Allograft
Emery et al.[32]	1998	45	55	58(27–88)		69M/38F		>2 years		Autograft	Autograft
Swank et al.[33]	1997	38	26	51(30–78)		37M/27F		39m(12–81m)		Allograft	Allograft
Yonenobu et al.[34]	1985	50	21	51.4±8.6	52.8±8.5	46M/4F	20M/1F	54m(14–157m)	30m(12–88m)	Autograft	Autograft

### 2.5 Statistical analysis

All statistical tests were performed using the Review Manager software (RevMan Version 5.1; The Cochrane Collaboration, Copenhagen, Denmark). Assessment for statistical heterogeneity was done using the Chi-squared and I-squared tests [16]. Values of I^2^ greater than 50% were considered to indicate substantial heterogeneity. A probability of *p*<0.05 was considered to be statistically significant. The results were expressed in terms of mean difference (MD) and 95% CI for continuous outcomes and in terms of odds ratio (OR) and 95% confidence interval (95% CI) for dichotomous outcomes. A random effects model was used for heterogeneous data; otherwise, a fixed effect model was used. Collected data were checked and entered into the computer by the two reviewers (Han and Liu).

## Results

### 3.1 Search results

**Figure 1:**
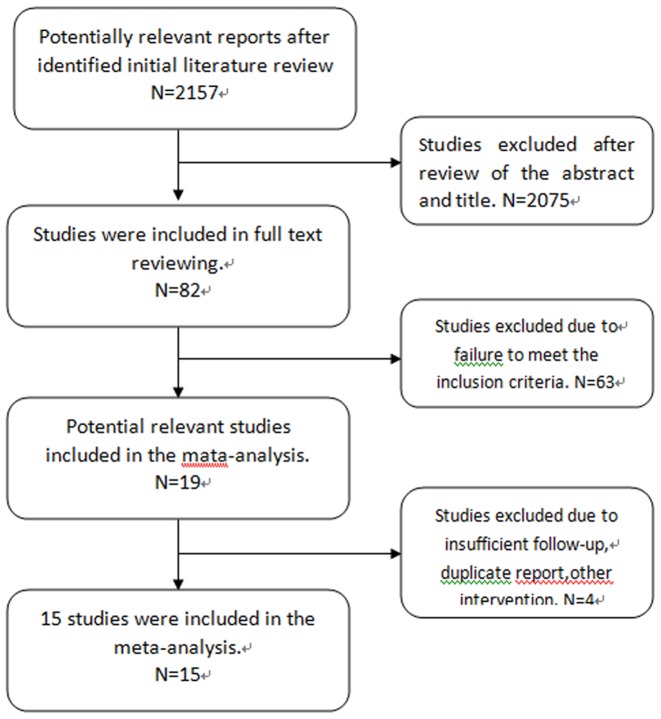
Flow diagram detailing study inclusion.

Initial electronic database searches yielded 2157 relevant titles. Of these, 2075 were excluded after review of the abstract and title for being unrelated to the topic at hand, not human studies, not comparative studies, or for being case reports or review articles. A further 63 studies were subsequently excluded due to failure to meet the inclusion criteria after review of the full text. One article were excluded due to insufficient follow-up [17]. An additional two studies were excluded due to other interventions [18], [19]. Two articles identified were written by the same author [20], [21], and we selected the one most recently published [21]. As a result, fifteen studies fulfilled the eligibility criteria [10], [21]–[34]. Study inclusion is detailed in *Fig 1*. A meta-analysis was conducted using these fifteen studies.

### 3.2 Demographic characteristics and quality assessment

The demographic characteristics of the included studies are presented in *Tables 1* *–* *3*. The 15 studies included a total of 1372 patients: 734 who underwent ACDF and 638 who underwent ACCF with various grafts, including autografts, allografts, and cage and/or plate systems. All participants in the fifteen studies had undergone follow-up for at least 2 years. No randomized controlled trials were identified; all fifteen studies included were retrospective studies. The quality of each study was assessed using the Newcastle Ottawa Quality Assessment Scale (NOQAS). This scale for non-randomized case controlled studies and cohort studies was used to allocate a maximum of nine points for the quality of selection, comparability, exposure, and outcomes for study participants. Of the studies, six scored 8 points and nine scored 7 points. Hence, the studies were of a relatively high quality (*Table 4*).

**Table 2:** Details and heterogeneity of clinical outcome measurement of the included studies.

Study	Hospital Stay	Operation Time	Blood Loss	JOA	VAS	NDI	Odom Criteria	Fusion Rate	Cobb angles of C2–C7	Segmental angle	Complications
**Li et al.** [22]	NA	NA	NA	YES	NA	NA	NA	YES	YES	NA	NA
**Liu et al.** [21]	NA	YES	YES	YES	NA	YES	YES	YES	YES	YES	YES
**Kyung et al.** [23]	YES	YES	YES	YES	YES	NA	NA	YES	YES	NA	YES
**Lin et al.** [24]	NA	YES	YES	YES	NA	YES	YES	YES	NA	YES	YES
**Guo et ai.** [25]	NA	YES	YES	YES	NA	NA	NA	YES	YES	YES	YES
**Park et al.** [26]	NA	NA	NA	NA	NA	NA	NA	NA	YES	NA	NA
**Oh et al.** [10]	YES	YES	YES	YES	YES	NA	NA	YES	YES	NA	NA
**Uribe et al.** [27]	NA	YES	YES	NA	NA	NA	YES	YES	NA	NA	YES
**Hwang et al.** [28]	YES	NA	NA	YES	YES	NA	NA	YES	YES	NA	YES
**Nirala et al.** [29]	NA	NA	NA	NA	NA	NA	YES	YES	NA	NA	YES
**Hilibrand et al.** [30]	NA	NA	NA	NA	NA	NA	YES	YES	NA	NA	US
**Jeffrey et al.** [31]	NA	NA	NA	NA	NA	NA	YES	YES	NA	NA	US
**Emery et al.** [32]	NA	NA	NA	NA	NA	NA	NA	YES	NA	NA	US
**Swank et al.** [33]	NA	NA	NA	NA	NA	NA	YES	NA	NA	NA	US
**Yonenobu et al.** [34]	NA	NA	NA	NA	NA	NA	NA	NA	NA	NA	YES
**Number of patients involved**	133	446	446	517	133	228	805	1140	432	295	749

**Table 3:** Details of complications of the included studies.

Study	Dysphagia	Hoarseness	C5 palsy	Infection	Cerebral fluid leakage	Donor site pain	Epidural hematoma	Graft related	Hardware related
**Liu et al.** [21]	YES	YES	YES	YES	YES	NA	YES	YES	YES
**Kyung et al.** [23]	YES	YES	NA	NA	YES	YES	NA	YES	YES
**Lin et al.** [24]	YES	YES	YES	NA	YES	NA	YES	YES	NA
**Guo et ai.** [25]	NA	NA	NA	NA	YES	NA	YES	NA	YES
**Uribe et al.** [27]	NA	NA	NA	NA	NA	NA	NA	YES	NA
**Hwang et al.** [28]	YES	YES	NA	NA	NA	YES	NA	NA	NA
**Nirala et al.** [29]	YES	YES	NA	YES	NA	YES	NA	YES	NA
**Yonenobu et al.** [34]	NA	NA	NA	YES	NA	NA	YES	YES	NA
**Number of patients involved**	531	531	228	380	335	303	366	620	215

**Table 4:** Quality assessment according to the Newcastle–Ottawa scale of the included studies.

Study	Selection	Comparability	Exposure	Total score
Li et al. [22]	3	2	3	8
Liu et al. [21]	3	2	3	8
Kyung et al. [23]	3	2	3	8
Lin et al. [24]	3	2	3	8
Guo et ai.[25]	3	2	3	8
Park et al. [26]	2	2	3	7
Oh et al. [10]	2	2	3	7
Uribe et al. [27]	2	2	3	7
Hwang et al. [28]	3	2	3	8
Nirala et al. [29]	2	2	3	7
Hilibrand et al. [30]	2	2	3	7
Jeffrey et al. [31]	2	2	3	7
Emery et al. [32]	2	2	3	7
Swank et al. [33]	2	2	3	7
Yonenobu et al. [34]	2	2	3	7

### 3.3 Clinical outcome analysis

#### 3.3.1 Hospital stay, blood loss and operation time

**Figure 2:**
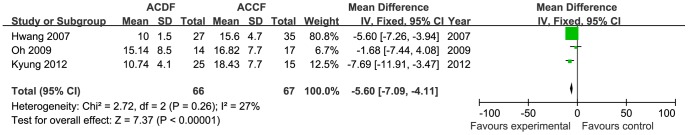
The forest plot for hospital stay between ACDF group and ACCF group, CI  =  confidence interval, df  =  degrees of freedom, IV  =  independent variable,SD  =  standard deviation.

**Figure 3:**
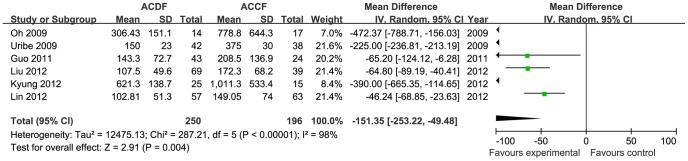
The forest plot for blood loss between ACDF group and ACCF group, CI  =  confidence interval, df  =  degrees of freedom, IV  =  independent variable, SD  =  standard deviation.

**Figure 4:**
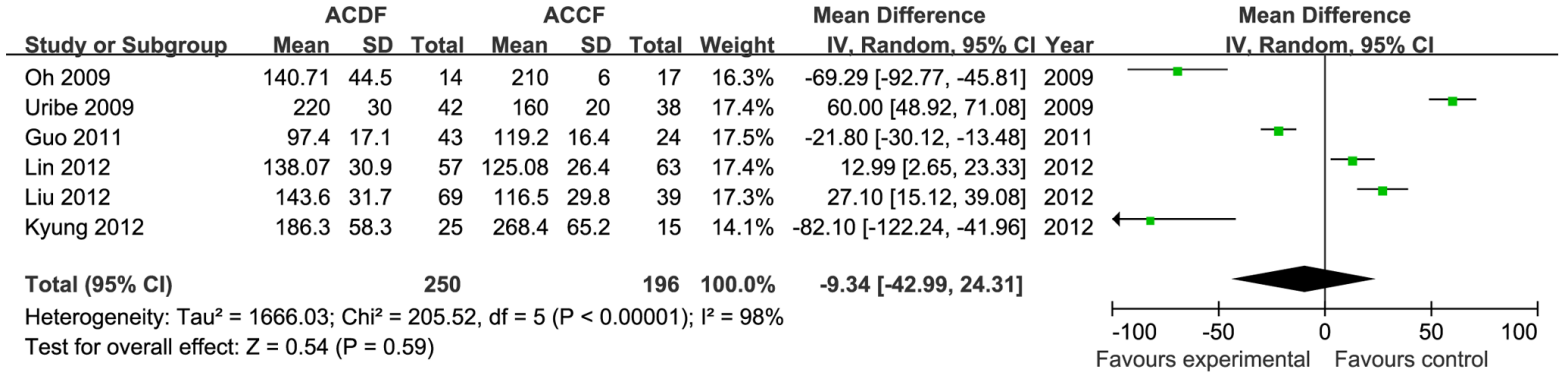
The forest plot for operation time between ACDF group and ACCF group, CI  =  confidence interval, df  =  degrees of freedom, IV  =  independent variable,SD  =  standard deviation.

Three studies were selected for the meta-analysis for hospital stay [10], [23], [27]. A total of 133 patients from 3 studies (66 patients for ACDF and 67 patients for ACCF) were included in this comparison. The available data demonstrated low heterogeneity (I^2^ = 27%). The hospital stay in the ACDF group was superior to the ACCF group (MD = −5.60, 95% CI = −7.09 to −4.11; *p*<0.00001; *Fig 2*). Six studies reported intraoperative blood loss and operation time; a total of 446 patients from 6 studies (250 patients for ACDF and 196 patients for ACCF) were included. Blood loss was significantly higher in the ACCF group compared with ACDF (MD = −151.35, 95% CI = −253.22 to −49.48; *p* = 0.004; *Fig 3*). There was no significant difference in operation time between the two treatment groups (MD = −9.34, 95% CI = −42.99 to 24.31; *p* = 0.59; *Fig 4*). There was significant heterogeneity in blood loss and operation time between the studies (heterogeneity: I^2^ = 98%), which can not be explained by our predefined subgroup analysis. Therefore, the quality of evidence for this outcome is low.

#### 3.3.2 JOA, VAS, NDI and Odom criteria

**Figure 5:**
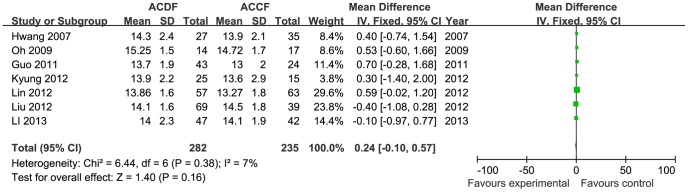
The forest plot for JOA score between ACDF group and ACCF group, CI  =  confidence interval, df  =  degrees of freedom, IV  =  independent variable, SD  =  standard deviation, JOA =  the Japanese Orthopedic Association scores.

**Figure 6:**
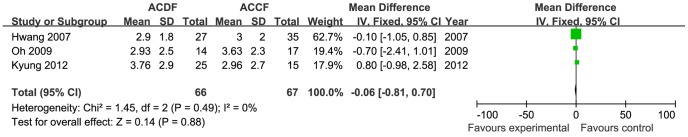
The forest plot for VAS score between ACDF group and ACCF group, CI  =  confidence interval, df  =  degrees of freedom, IV  =  independent variable,SD  =  standard deviation, VAS =  visual analogue score of neck.

**Figure 7:**
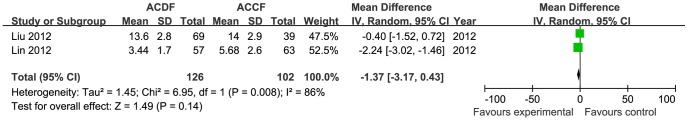
The forest plot for NDI score between ACDF group and ACCF group, CI  =  confidence interval, df  =  degrees of freedom, IV  =  independent variable,SD  =  standard deviation, NDI =  neck disability index.

**Figure 8:**
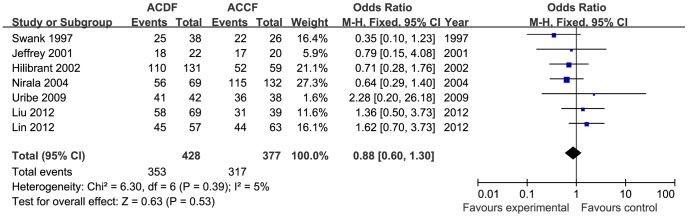
The forest plot for Odom's criteria between ACDF group and ACCF group,CI = confidence interval, df = degrees of freedom,MH = Mantel-Haenszel statistical method, SD  =  standard deviation.

The clinical outcome for 571 patients from seven studies was assessed using the JOA score (282 in the ACDF group and 235 in the ACCF group). There was no significant difference in the final follow-up JOA score between the two groups (MD = 0.24, 95% CI = −0.10 to 0.57; *p* = 0.16), with low heterogeneity (I^2^ = 7%; *Fig 5*). Three studies included reports of neck pain in the VAS scores (66 in the ACDF group and 67 in the ACCF group). There was no difference in neck VAS score between ACDF and ACCF (MD = −0.06, 95% CI = −0.81to 0.70; *p* = 0.88) with no heterogeneity (I^2^ = 0%; *Fig 6*). Two studies reported a final follow-up NDI score; there was no significant difference between the two treatment groups (MD = −1.37, 95% CI = −3.17 to 0.43; *p* = 0.14; *Fig 7*). Seven trials reported the Odom criteria (428 in the ACDF group and 377 in the ACCF group). The patients with excellent or good clinical outcomes were similar in the two groups (OR = 0.88, 95% CI = 0.60 to 1.30; *p* = 0.53) and the available data demonstrated low heterogeneity (I^2^ = 5%; *Fig 8*).

#### 3.3.3 Radiographic assessment (fusion rate, Cobb angle of C2–C7, and segmental angle)

**Figure 9:**
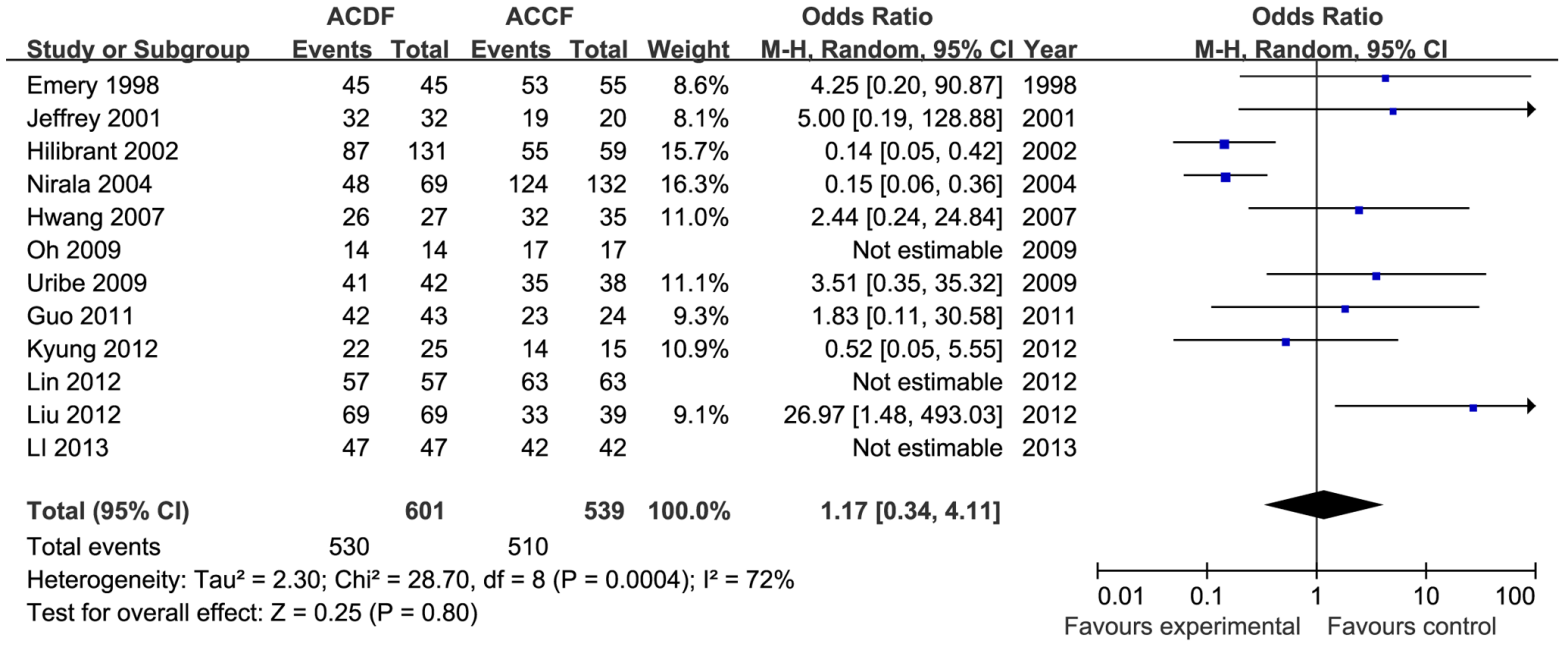
The forest plot for fusion rate between ACDF group and ACCF group,CI = confidence interval, df = degrees of freedom,MH = Mantel-Haenszel statistical method, SD  =  standard deviation.

**Figure 10:**
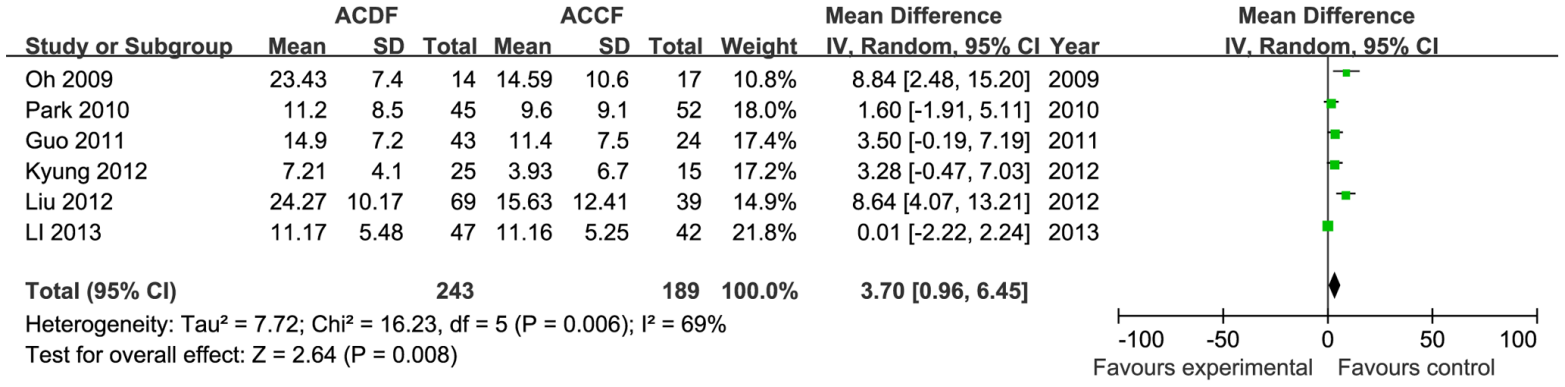
The forest plot for cobb angles of C2–C7 score between ACDF group and ACCF group, CI  =  confidence interval, df  =  degrees of freedom, IV  =  independent variable,SD  =  standard deviation.

**Figure 11:**
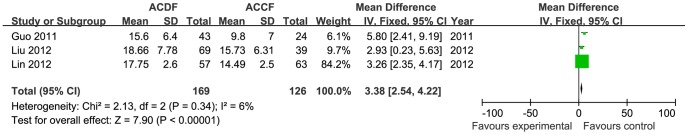
The forest plot for cobb angles of segmental between ACDF group and ACCF group, CI  =  confidence interval, df  =  degrees of freedom, IV  =  independent variable,SD  =  standard deviation.

Twelve studies used radiographs to assess the consolidation of the fusion (n = 1140 patients, 601 in the ACDF group and 539 in the ACCF group). The incidence of fusion was not different between the two groups (OR = 1.17, 95% CI = 0.34 to 4.11; *p* = 0.80), with moderate heterogeneity (I^2^ = 72%; *Fig 9*). Six trials reported the Cobb angle of C2–C7 (243 in the ACDF group and 189 in the ACCF group) and three trials reported the segmental angle (169 in the ACDF group and 126 in the ACCF group). Statistical analysis showed significant differences between the ACDF and ACCF groups for changes of the angle of C2–C7 (MD = 3.70, 95% CI = 0.96 to 6.45; *p* = 0.008; I^2^ = 69%; *Fig 10*) and the segmental angle (MD = 3.38, 95% CI = 2.54 to 4.22; *p*<0.00001; I^2^ = 6%; *Fig 11*).

#### 3.3.4 Complications

**Figure 12:**
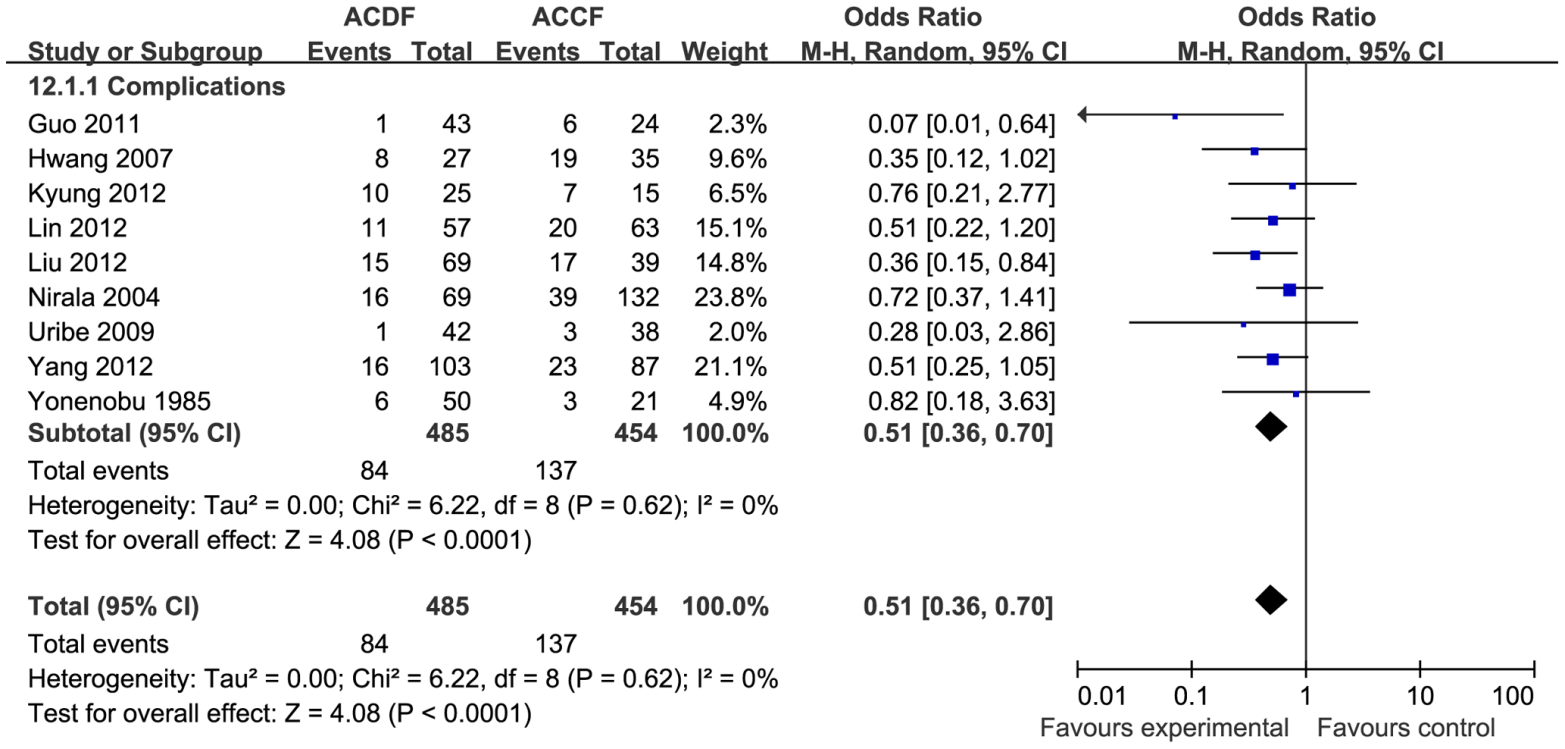
The forest plot for totally complications between ACDF group and ACCF group,CI = confidence interval, df = degrees of freedom,MH = Mantel-Haenszel statistical method, SD  =  standard deviation.

**Figure 13:**
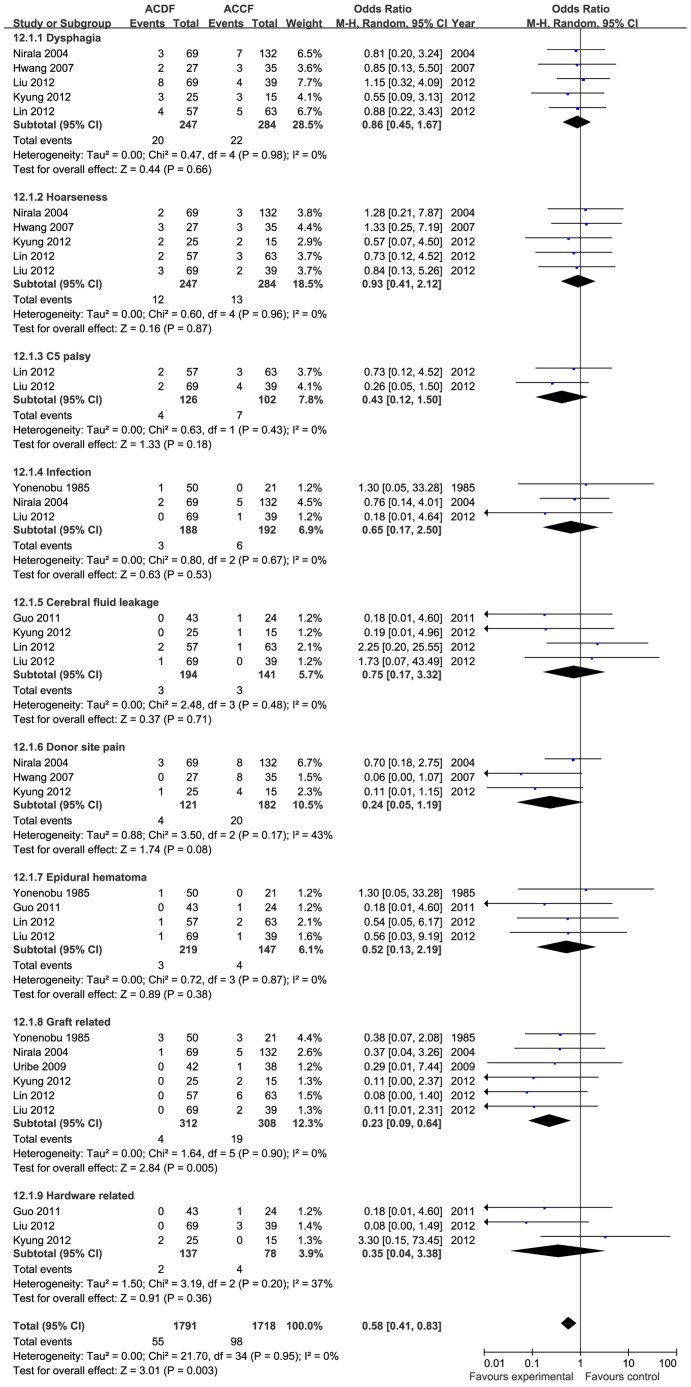
The forest plot for the subgroups of complication between ACDF group and ACCF group,CI = confidence interval, df = degrees of freedom,MH = Mantel-Haenszel statistical method, SD  =  standard deviation.

Eight studies reported complications (n = 749 patients, 382 in the ACDF group and 367 in the ACCF group), however, the records of post-operative complications were variable. Some studies described all complications, whereas some provided only the major complications. The incidence of complications was significantly higher in the ACCF group than in the ACDF group (OR = 0.50, 95% CI = 0.35 to 0.73; *p* = 0.0003), with no heterogeneity (I^2^ = 0%; *Fig 12*). There was a significant difference in graft-related complications, however, there were no differences in dysphagia, hoarseness, C5 palsy, infection, cerebral fluid leakage, donor site pain, epidural hematoma, or hardware-related complications (*Fig 13*).

## Discussion

Although the surgical treatment for cervical spondylotic myelopathy (CSM) has a history going back sixty years, the selection of surgical procedures remains controversial and challenging. The common surgical procedures used include the anterior, posterior, and combined anteroposterior approaches. Anterior approaches to the cervical spine are recognized as a reliable and effective method to treat CSM and they have been widely accepted as an appropriate operative procedure. Anterior decompression and fusion can remove the compressive pathology and reconstruct the alignment of the cervical spine, yielding good clinical results. The type of decompression and reconstruction technique are the two important decisions to be made. Anterior decompression and fusion include cervical discectomy with fusion (ACDF) and anterior cervical corpectomy with fusion (ACCF) [35]–[37], however, the ideal anterior decompression method is controversial.

Although some relevant studies comparing the ACDF and ACCF have been reported, the evidence regarding whether ACDF is superior to ACCF remains insufficient, owing to ambiguous results. We therefore conducted a meta-analysis to determine whether ACDF is associated with better clinical outcomes compared with ACCF. In this meta-analysis, we used strict eligibility criteria. Although no RCT studies were included in our study, all selected studies were of high quality according to the Newcastle Ottawa Quality Assessment Scale (NOQAS) and the baseline variables were similar. Thus, we considered the included reports suitable for meta-analysis. Clinical outcomes (hospital stay, JOA, VAS, NDI score and Odom criteria), surgical outcomes (operation time, blood loss, and perioperative complications), and radiographic outcomes (rate of fusion, Cobb angle of C2–C7, and segmental angle) were assessed in the meta-analysis.

There was a significant difference in hospital stay between ACDF and ACCF. A shorter hospital stay makes ACDF a better proposition than ACCF. In the meta-analysis of JOA, VAS and NDI, scores were similar in the two groups. However, both groups demonstrated a significant post-operative increase in JOA scores and decrease in VAS scores, an increase that was maintained at the final follow-up. There was also no difference in Odom criteria between the two groups. These findings indicate that both groups achieved adequate decompression of the spinal cord and nerve roots that were compressed by herniated discs or osteophytes, and that these patients benefited from reconstruction of the spinal column.

In the meta-analysis, operation time and blood loss were selected to evaluate surgical trauma. Both the overall and subgroup analyses revealed that although blood loss was significantly higher in the ACCF group than in the ACDF group, the operation time was similar in the two groups. This indicates that, in the treatment of CSM, the surgical trauma associated with ACCF is higher than with ACDF. We selected the total complications for meta-analysis to evaluate complication-related outcomes, and found a higher incidence of complications with ACCF than with ACDF (OR = 0.50, 95% CI = 0.35 to 0.73, *p* = 0.0003). Subgroup analysis observed apart from graft related complications is significantly higher in the ACCF group (*p* = 0.005), while other subgroups, namely dysphagia, hoarseness, C5 palsy, infection, cerebral fluid leakage, donor site pain, epidural hematoma and hardware-related complications, were similar between the two groups. There was no heterogeneity between the two groups for total complications for all subgroups (I^2^ = 0). Considering the most significantly different complications were graft-related, this seemed to be due to technical reasons. Some authors consider that ACDF offers more fixation points to hold the construct rigidly in place, but ACCF provide only two points of fixation. The lack of fixation points may therefore be the reason for the higher graft-related complication rates in the ACCF group [15], [38]. There were similar rates of dysphagia and hoarseness between the two groups in this meta-analysis; and they are the most common sequelae. Some studies have reported that post-operative dysphagia occurs in 2–48% of patients [39] and post-operative hoarseness occurs in 3–11% of patients [40], [41], but these symptoms are frequently transient. The etiology of dysphagia may be multifactorial, including hematoma formation and prolonged retraction and denervation of the upper esophagus by injury to the pharyngeal plexus [42]. The etiology of post-operative hoarseness has been postulated to be related to direct injury to the recurrent or superior laryngeal nerves.

Regarding the fusion rate, in the current meta-analysis, patients who underwent ACDF were not significantly different from those who underwent ACCF (*p* = 0.63). However, some studies have reported that ACDF has a high rate of non-union, because they consider that ACCF can not only easily resolve retro-vertebral compressive pathology but also reduce the graft-host interface [23], [25], [29], [30]. However, meta-analysis is a statistical analysis of data collected from several different studies on the same problem, pooling outcomes in order to arrive at a more unbiased and scientific conclusion [43], [44], so we regard the fusion rate to be similar between the two groups. In this meta-analysis, both the ACDF and ACCF groups had significantly increased lordosis of C2–C7 and fusion segments, but the increase was greater in the ACDF group than in those with ACCF. Some studies have reported that ACDF can provide multiple points of distraction and fixation in addition to the graft and interbody space shaping, and can also restore alignment by pulling the involved vertebral bodies toward the lordotic ventral plate. However, ACCF grafts may straighten the cervical spinal column between the remaining vertebral bodies [24].

There are several limitations to this meta-analysis. Firstly, none of the studies included in the meta analysis were RCTs. Secondly, there was a variable length of follow-up between the studies and this is particularly important for evaluating surgery results. Thirdly, clinical heterogeneity might be caused by the various indications for surgery and the surgical technologies used at the different treatment centers. Finally, these studies lack a gold standard outcome to evaluate the post-operative clinical effect.

### Conclusion

Based on a systematic review of the literature and meta-analysis of ACDF and ACCF for the treatment of CSM, the following conclusions may be drawn. The clinical outcomes of ACDF are superior to ACCF for hospital stay, blood loss, complications and increased cervical lordosis, but the outcomes of operation time, fusion rate, Odom criteria and JOA, VAS, and NDI scores are equivalent between the two groups. This meta-analysis highlights the surgical and outcome differences between ACDF and ACCF in the treatment of CSM. Due to the varying pathoanatomy of spinal cord compression leading to CSM, individualized treatment decisions should be based upon the location of the compressive pathology. If significant retrovertebral compression on the spinal cord is present then ACCF is the preferred treatment. In the absence of significant retrovertebral disease, ACDF is the preferred treatment. However future studies with high methodological quality and long-term follow-up periods are needed for updated meta-analyses, in order to better evaluate the two procedures for CSM treatment.

## Supporting Information

Checklist S1 **PRISMA Checklist.**
(DOC)

## References

[1] Carette S, Fehlings MG (2005) Clinical practice. Cervical radiculopathy. N Engl J Med 353: 392–399. 16049211 10.1056/NEJMcp043887

[2] Rao RD, Gourab K, David KS (2006) Operative treatment of cervical spondylotic myelopathy. J Bone Joint Surg Am 88: 1619–1640. 16818991 10.2106/JBJS.F.00014

[3] Ghogawala Z, Coumans JV, Benzel EC, Stabile LM, Barker 2nd FG (2007) Ventral versus dorsal decompression for cervical spondylotic myelopathy: surgeons' assessment of eligibility for randomization in a proposed randomized controlled trial: results of a survey of the Cervical Spine Research Society. Spine (Phila Pa 1976) 32: 429–436. 17304133 10.1097/01.brs.0000255068.94058.8a

[4] Toledano M, Bartleson JD (2013) Cervical spondylotic myelopathy. Neurol Clin 31: 287–305. 23186905 10.1016/j.ncl.2012.09.003

[5] Lebl DR, Hughes A, Cammisa Jr FP, O'Leary PF (2011) Cervical spondylotic myelopathy: pathophysiology, clinical presentation, and treatment. HSS J 7: 170–178. 22754419 10.1007/s11420-011-9208-1 PMC3145857

[6] Young WF (2000) Cervical spondylotic myelopathy: a common cause of spinal cord dysfunction in older persons. Am Fam Physician 62: : 1064–1070, 1073. 10997531

[7] Sypert GW, Cole HO (1999) Management of multilevel cervical spondylosis with myelopathy. Surg Neurol 51: 4–5. 9952115 10.1016/s0090-3019(97)00488-6

[8] Smith GW, Robinson RA (1958) The treatment of certain cervical-spine disorders by anterior removal of the intervertebral disc and interbody fusion. J Bone Joint Surg Am 40-A: 607–624. 13539086

[9] Cloward RB (1958) The anterior approach for removal of ruptured cervical disks. J Neurosurg 15: 602–617. 13599052 10.3171/jns.1958.15.6.0602

[10] Oh MC, Zhang HY, Park JY, Kim KS (2009) Two-level anterior cervical discectomy versus one-level corpectomy in cervical spondylotic myelopathy. Spine (Phila Pa 1976) 34: 692–696. 19333101 10.1097/BRS.0b013e318199690a

[11] Fountas KN, Kapsalaki EZ, Nikolakakos LG, Smisson HF, Johnston KW, et al (2007) Anterior cervical discectomy and fusion associated complications. Spine (Phila Pa 1976) 32: 2310–7. 17906571 10.1097/BRS.0b013e318154c57e

[12] Gore DR (2001) The arthrodesis rate in multilevel anterior cervical fusions using autogenous fibula. Spine (Phila Pa 1976) 26: 1259–1263. 11389393 10.1097/00007632-200106010-00016

[13] Wang JC, Hart RA, Emery SE, Bohlman HH (2003) Graft migration or displacement after multilevel cervical corpectomy and strut grafting. Spine (Phila Pa 1976) 28: 1016–1021 discussion 1021–1012. 12768141 10.1097/01.BRS.0000061998.62204.D7

[14] Hee HT, Majd ME, Holt RT, Whitecloud 3rd TS, Pienkowski D (2003) Complications of multilevel cervical corpectomies and reconstruction with titanium cages and anterior plating. J Spinal Disord Tech 16: 1–8 discussion 8–9. 12571477 10.1097/00024720-200302000-00001

[15] Vaccaro AR, Falatyn SP, Scuderi GJ, Eismont FJ, McGuire RA, et al (1998) Early failure of long segment anterior cervical plate fixation. J Spinal Disord 11: 410–415. 9811102

[16] Higgins JP, Thompson SG (2002) Quantifying heterogeneity in a meta-analysis. Stat Med 21: 1539–1558. 12111919 10.1002/sim.1186

[17] Burkhardt JK, Mannion AF, Marbacher S, Dolp PA, Fekete TF, et al (2013) A comparative effectiveness study of patient-rated and radiographic outcome after 2 types of decompression with fusion for spondylotic myelopathy: anterior cervical discectomy versus corpectomy. Neurosurg Focus 35: E4. 10.3171/2013.3.FOCUS1396 23815249

[18] Hilibrand AS, Fye MA, Emery SE, Palumbo MA, Bohlman HH (2001) Impact of smoking on the outcome of anterior cervical arthrodesis with interbody or strut-grafting. J Bone Joint Surg Am 83-A: 668–673. 11379735 10.2106/00004623-200105000-00004

[19] Lee MJ, Bazaz R, Furey CG, Yoo J (2007) Risk factors for dysphagia after anterior cervical spine surgery: a two-year prospective cohort study. Spine J 7: 141–147. 17321961 10.1016/j.spinee.2006.02.024

[20] Liu Y, Qi M, Chen H, Yang L, Wang X, et al (2012) Comparative analysis of complications of different reconstructive techniques following anterior decompression for multilevel cervical spondylotic myelopathy. Eur Spine J 21: 2428–2435. 22644433 10.1007/s00586-012-2323-y PMC3508223

[21] Liu Y, Hou Y, Yang L, Chen H, Wang X, et al (2012) Comparison of 3 reconstructive techniques in the surgical management of multilevel cervical spondylotic myelopathy. Spine (Phila Pa 1976) 37: E1450–1458. 22869063 10.1097/BRS.0b013e31826c72b4

[22] Li J, Zheng Q, Guo X, Zeng X, Zou Z, et al (2013) Anterior surgical options for the treatment of cervical spondylotic myelopathy in a long-term follow-up study. Arch Orthop Trauma Surg 133: 745–751. 23503888 10.1007/s00402-013-1719-4

[23] Song KJ, Lee KB, Song JH (2012) Efficacy of multilevel anterior cervical discectomy and fusion versus corpectomy and fusion for multilevel cervical spondylotic myelopathy: a minimum 5-year follow-up study. Eur Spine J 21: 1551–1557. 22526699 10.1007/s00586-012-2296-x PMC3535261

[24] Lin Q, Zhou X, Wang X, Cao P, Tsai N, et al (2012) A comparison of anterior cervical discectomy and corpectomy in patients with multilevel cervical spondylotic myelopathy. Eur Spine J 21: 474–481. 21826497 10.1007/s00586-011-1961-9 PMC3296841

[25] Guo Q, Bi X, Ni B, Lu X, Chen J, et al (2011) Outcomes of three anterior decompression and fusion techniques in the treatment of three-level cervical spondylosis. Eur Spine J 20: 1539–1544. 21448583 10.1007/s00586-011-1735-4 PMC3175896

[26] Park Y, Maeda T, Cho W, Riew KD (2010) Comparison of anterior cervical fusion after two-level discectomy or single-level corpectomy: sagittal alignment, cervical lordosis, graft collapse, and adjacent-level ossification. Spine J 10: 193–199. 19850532 10.1016/j.spinee.2009.09.006

[27] Uribe JS, Sangala JR, Duckworth EA, Vale FL (2009) Comparison between anterior cervical discectomy fusion and cervical corpectomy fusion using titanium cages for reconstruction: analysis of outcome and long-term follow-up. Eur Spine J 18: 654–662. 19214597 10.1007/s00586-009-0897-9 PMC3234010

[28] Hwang SL, Lee KS, Su YF, Kuo TH, Lieu AS, et al (2007) Anterior corpectomy with iliac bone fusion or discectomy with interbody titanium cage fusion for multilevel cervical degenerated disc disease. J Spinal Disord Tech 20: 565–570. 18046168 10.1097/BSD.0b013e318036b463

[29] Nirala AP, Husain M, Vatsal DK (2004) A retrospective study of multiple interbody grafting and long segment strut grafting following multilevel anterior cervical decompression. Br J Neurosurg 18: 227–232. 15327222 10.1080/02688690410001732643

[30] Hilibrand AS, Fye MA, Emery SE, Palumbo MA, Bohlman HH (2002) Increased rate of arthrodesis with strut grafting after multilevel anterior cervical decompression. Spine (Phila Pa 1976) 27: 146–151. 11805659 10.1097/00007632-200201150-00005

[31] Wang JC, McDonough PW, Endow KK, Delamarter RB (2001) A comparison of fusion rates between single-level cervical corpectomy and two-level discectomy and fusion. J Spinal Disord 14: 222–225. 11389372 10.1097/00002517-200106000-00006

[32] Emery SE, Bohlman HH, Bolesta MJ, Jones PK (1998) Anterior cervical decompression and arthrodesis for the treatment of cervical spondylotic myelopathy. Two to seventeen-year follow-up. J Bone Joint Surg Am 80: 941–951. 9697998 10.2106/00004623-199807000-00002

[33] Swank ML, Lowery GL, Bhat AL, McDonough RF (1997) Anterior cervical allograft arthrodesis and instrumentation: multilevel interbody grafting or strut graft reconstruction. Eur Spine J 6: 138–143. 9209883 10.1007/BF01358747 PMC3454584

[34] Yonenobu K, Fuji T, Ono K, Okada K, Yamamoto T, et al (1985) Choice of surgical treatment for multisegmental cervical spondylotic myelopathy. Spine (Phila Pa 1976) 10: 710–716. 4081877 10.1097/00007632-198510000-00004

[35] Grob D, Luca A (2010) Surgery for cervical stenosis: anterior cervical decompression, corpectomy, and fusion. Eur Spine J 19: 1801–1802. 20960656 10.1007/s00586-010-1571-y PMC7571852

[36] Dean CL, Gabriel JP, Cassinelli EH, Bolesta MJ, Bohlman HH (2009) Degenerative spondylolisthesis of the cervical spine: analysis of 58 patients treated with anterior cervical decompression and fusion. Spine J 9: 439–446. 19111509 10.1016/j.spinee.2008.11.010

[37] Ba Z, Zhao W, Wu D, Shen B, Yu B, et al (2012) Box cages packed with local decompression bone were efficient in anterior cervical discectomy and fusion: five- to 10-year follow-up. Spine (Phila Pa 1976) 37: E1260–1263. 22744617 10.1097/BRS.0b013e318265df75

[38] Sasso RC, Ruggiero Jr RA, Reilly TM, Hall PV (2003) Early reconstruction failures after multilevel cervical corpectomy. Spine (Phila Pa 1976) 28: 140–142. 12544930 10.1097/00007632-200301150-00009

[39] Clements DH, O'Leary PF (1990) Anterior cervical discectomy and fusion. Spine (Phila Pa 1976) 15: 1023–1025. 2263966 10.1097/00007632-199015100-00008

[40] Apfelbaum RI, Kriskovich MD, Haller JR (2000) On the incidence, cause, and prevention of recurrent laryngeal nerve palsies during anterior cervical spine surgery. Spine (Phila Pa 1976) 25: 2906–2912. 11074678 10.1097/00007632-200011150-00012

[41] Heeneman H (1973) Vocal cord paralysis following approaches to the anterior cervical spine. Laryngoscope 83: 17–21. 4683910 10.1288/00005537-197301000-00002

[42] Welsh LW, Welsh JJ, Chinnici JC (1987) Dysphagia due to cervical spine surgery. Ann Otol Rhinol Laryngol 96: 112–115. 3813373 10.1177/000348948709600125

[43] Dickman CA, Yahiro MA, Lu HT, Melkerson MN (1994) Surgical treatment alternatives for fixation of unstable fractures of the thoracic and lumbar spine. A meta-analysis. Spine (Phila Pa 1976) 19: 2266S–2273S. 7817241 10.1097/00007632-199410151-00003

[44] Haher TR, Merola A, Zipnick RI, Gorup J, Mannor D, et al (1995) Meta-analysis of surgical outcome in adolescent idiopathic scoliosis. A 35-year English literature review of 11,000 patients. Spine (Phila Pa 1976) 20: 1575–1584. 7570172 10.1097/00007632-199507150-00005

